# The decade of exosomal long RNA species: an emerging cancer antagonist

**DOI:** 10.1186/s12943-018-0823-z

**Published:** 2018-03-20

**Authors:** Ruihao Zhou, Kaddie Kwok Chen, Jingtao Zhang, Bufan Xiao, Zhaohao Huang, Cheng Ju, Jun Sun, Feifei Zhang, Xiao-Bin Lv, Guofu Huang

**Affiliations:** 1 Nanchang Key Laboratory of Cancer Pathogenesis and Translational Research, Center Laboratory, The Third Affiliated Hospital of Nanchang University, Nanchang, Jiangxi 330008 People’s Republic of China; 2 The First Clinical Department, Medical School of Nanchang University, Nanchang, Jiangxi 330008 People’s Republic of China; 3 Cornell University, Ithaca, NY 14853 USA; 4 Department of Ophthalmology, The Third Affiliated Hospital of Nanchang University, Nanchang, Jiangxi 330008 People’s Republic of China; 5 Department of Cardiothoracic Surgery, The First Affiliated Hospital of Nanchang University, Nanchang, Jiangxi 330008 People’s Republic of China

**Keywords:** Exosome, mRNA, lncRNA, circRNA, esRNA, Cancer biology, Tumor formation and progression

## Abstract

Exosomes have emerged as a novel approach for the treatment and diagnosis of cancer after RNA content was discovered in exosomes in 2007. As important meditators of intercellular communication, exosomes have become a strong focus of investigation for researchers in the past decade, as witnessed through the exponential increase of research on exosomes. The capability of exosomes to transfer functionally active cargo highlights their importance as promising biomarkers and diagnostic molecules, as well as prospective drug delivery systems. The accessibility of exosomes in nearly all biofluids additionally alludes to its unprecedented ability in various types of cancers due to its extensive impact on tumor formation and progression. This review analyzes the role of exosomal long RNA species, which is comprised of mRNA, lncRNA, and circRNA, in tumor formation and progression, with an emphasis on their potential as future diagnostic biomarkers and treatment vectors in cancer biology. Their alignment with the development of exosomal databases is further examined in this review, in view of the accumulation of studies published on exosomes in the past decade.

**Electronic supplementary material:**

The online version of this article (10.1186/s12943-018-0823-z) contains supplementary material, which is available to authorized users.

## Background

**Fig. 1:**
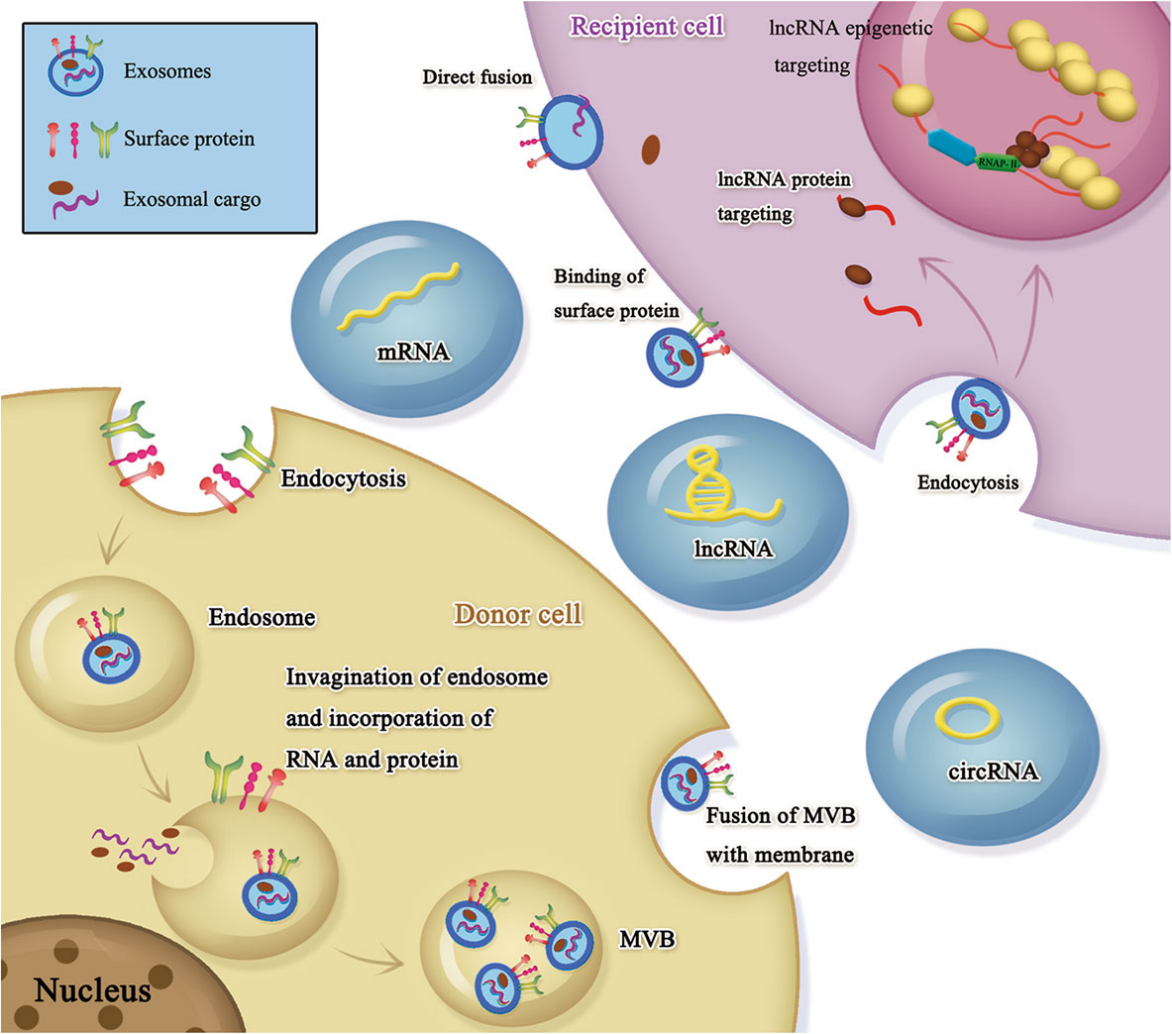
The biogenesis of exosomes. Beginning with endocytosis, the biogenesis of exosomes initially leads to the formation of endosomes. Further invagination of the endosomal membrane results in the incorporation of cytosolic protein or RNA within the endosome. The resulting multi-vesicular bodies (MVBs) then fuse with the plasma membrane and release the exosomes into the extracellular space, allowing the exosomes to interact with the recipient cells via endocytosis, direct fusion, or the binding of surface proteins. Once inside the recipient cells, RNA content, such as lncRNAs, can target proteins or epigenetic marks—affecting protein function and controlling the state of gene expression

Exosomes are currently best defined as small membranous vesicles that are released into the cell exterior upon fusion of the multi-vesicular body (MVB) with the cytoplasmic membrane. These vesicles are distinguished from other extracellular vesicles (EVs) by their size of 40-100 nm in diameter and specific surface molecular characteristics, namely the presence of tetraspanins such as CD9 and CD63 [1, 2]. EV cargo is comprised of specific contents that depend on the original cell type and condition from which they originated [3, 4]. Exosomal composition can further vary due to the selective sorting of cargo into exosomes [5]. In addition to multiple proteins, nucleic acids, and lipids that have been detected in exosomes, exosomes also contain messenger RNAs (mRNAs) and non-coding RNAs—such as microRNAs (miRNAs), long non-coding RNAs (lncRNAs), and circular RNAs (circRNAs) [6–9]. In the recent years, a large number of researchers have focused on evaluating exosomal miRNA content and characterizing its effect on various diseases. However, the lack in quantity and expression specificity greatly limit the value of miRNAs as diagnostic molecules. Other types of RNA, such as the long RNA species [10, 11], may play an equal or perhaps an even more significant role in cell-cell communication by altering biological signaling pathways that affect disease progression (Fig. 1). In this review, the long RNA species are defined as mRNAs, lncRNAs, and circRNAs larger than 200 nucleotides.

Exosomes are readily accessible in nearly all types of human biofluids—including saliva, breast milk, cerebrospinal fluid, ascites, urine, and semen—and are important conveyors of the immune response due to its widespread presence in the human body [12–18]. Its detection in many body fluids is also evidence of its stability in a variety of adverse environments. Subsequently, the function of exosomes has been extensively studied. Accumulating evidence has shown that exosomes are important molecules for cell-to-cell communication and are involved in many physiological and pathological processes, including cell migration, angiogenesis, immune response, and tumor cell growth [19, 20]. Exosomes were initially assumed to be released by cells as waste cargo when they were first discovered [21, 22]. However, they are now recognized as important sources of diagnostic biomarkers and carriers of information flow. Through their involvement in cell-to-cell communication, exosomes have been shown to transfer biologically active molecules to its recipient cell, thus altering the content and behavior of the recipient cell [4, 23]. As exosomes transfer functionally active cargo, the possibility of future treatments enlisting the help of exosomes to deliver therapeutic drugs to cancer cells is a topic of much discussion [24–31].

**Fig. 2:**
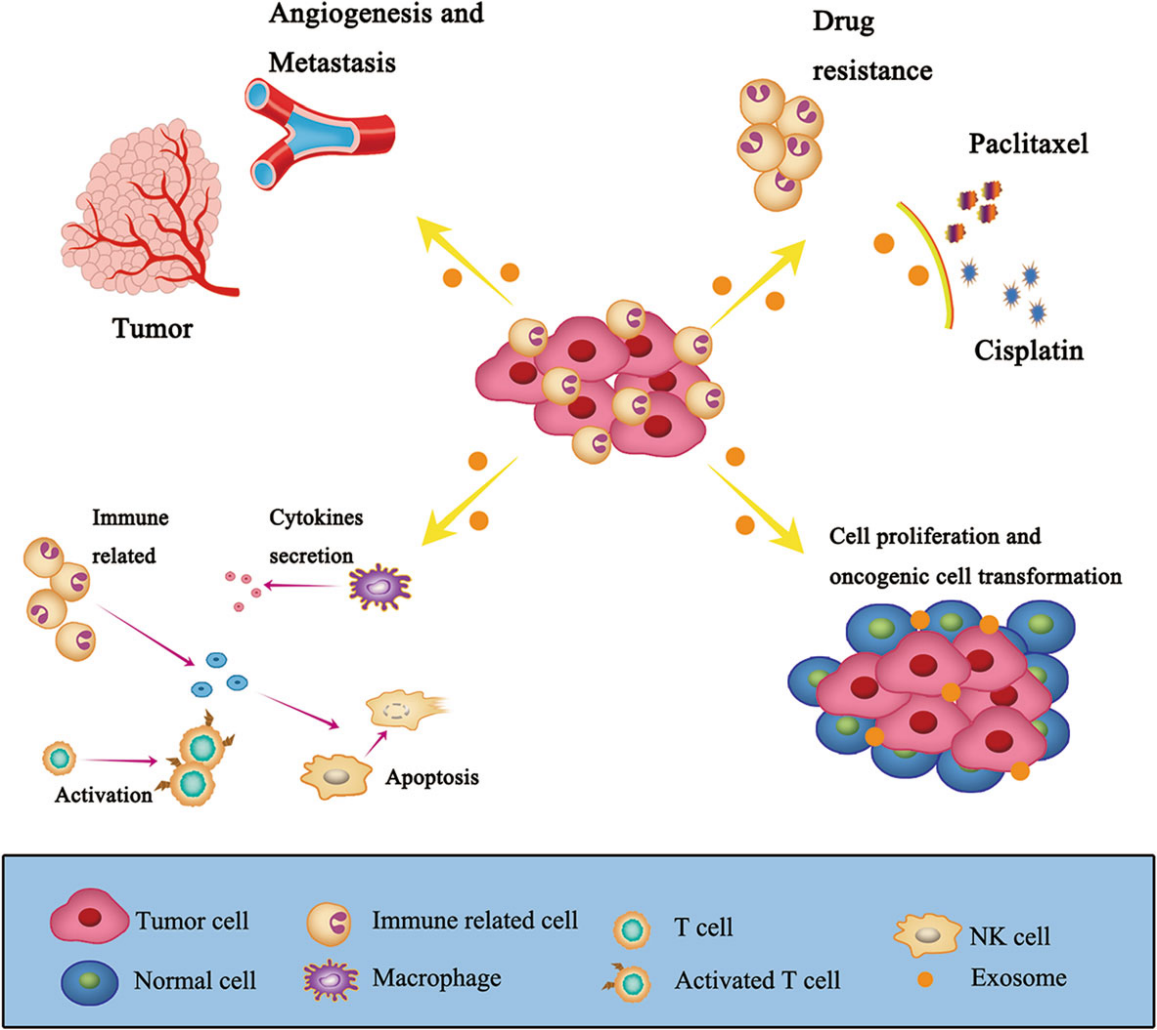
The role of exosomes in cancer biology. Due to their extensive effect on the tumor environment, released exosomes can promote angiogenesis and tumor metastasis, promote drug resistance, initiate immune responses, and advance cell proliferation and oncogenic cell transformation

Comprehension of the mechanisms involved in the interaction of tumor cells with its environment is essential for the understanding of cancer biology. Tumor-derived exosomes have been reported to play an important role in the development and prognosis of tumors, and can be used to alter tumor microenvironment, mediate tumor cell proliferation, angiogenesis, metastasis, and drug resistance (Fig. 2) [32, 33]. Intriguingly, researchers found a significant increase in the level of exosomes released by tumor cells as well as a contrast in exosomal content from normal physiological conditions [34, 35]. Tumor cells also have a precise targeting mechanism for the contents of exosomes, suggesting that exosomes play an important role in tumor formation and progression [36]. Consistent with the fact that exosomes are present in many body fluids, these factors make exosomes an attractive target for the discovery of new cancer biomarkers and therapeutic targets.

Herein, we summarize the role of exosome-derived RNAs in cancer, focusing on mRNAs, lncRNAs, and circRNAs. While miRNAs are important exosomal cargos, we focus on the potential of other RNAs in exosomes. The study of mRNAs, lncRNAs, and circRNAs in exosomes are relatively new in comparison to miRNAs and serve as promising sources of new therapeutic targets. Moreover, we analyze the role of exosome-derived RNA in tumor formation and progression in the context of its development in the past decade, with an emphasis on their potential as future diagnostic biomarkers and treatment vectors in cancer biology.

## Exosomes and exosome-derived RNA in cancer biology

Exosomes play a multifaceted role in the development and progression of tumors. In one aspect, exosomes have the ability to inhibit tumor cell growth. Normal cell-secreted exosomes can transfer tumor suppressor genes into cancerous cells and inhibit the proliferation of tumor cells by blocking the corresponding signaling pathways [37, 38]. Exosomes derived from tumor cells can induce specific, tumor resistance responses, such as the release of tumor-specific antigens by T cell exocytosis [39]. In another aspect, exosomes can promote the occurrence and development of tumors and play an important role in the tumor invasion and metastasis process. Such cancerous cell-derived exosomes can promote tumor growth and inhibit the cytotoxic effects of natural killer (NK) cells and T cells [40–42]. Tumor-derived exosomes can transfer pathologically expressed substances to normal cells in that the growth and differentiation of the latter are not inhibited [43, 44]. Due to its presence in various body fluids, exosomes can be also transported throughout the body via the same mechanism of action for achieving tumor metastasis [45]. In short, through its involvement in cell-to-cell transduction, tumor formation, and resistance to tumor progression, exosomes are a critical component of the tumor microenvironment.

Previous studies have reported that exosomes serve as protection against RNA degradation. Through a comparison of samples stored in different storage conditions, Hong et al. found that exosomes provide secure protection of its contents [46]. Exosomes contain and stabilize different amounts of RNA, which are transported to the target cells via endocytosis. Exosome-derived RNA is relatively stable throughout the process, affecting the phenotype and function of mRNA in the recipient cells. However, an important subject of consideration is whether the RNA molecules are intact or partially degraded [47]. Intact molecules can encode functional proteins and potentially retain original functions, whereas partially degraded molecules may acquire new functions—affecting cellular processes depending on their newly assumed functions.

The area of exosome-derived RNA has rapidly developed since it was initially reported in 2007, as reflected by the increase in publications in the past decade (Additional file 1). Previous studies have shown that the RNA profile of exosome-derived cells and its donor cells differs substantially [48, 49]. Total exosomal RNA typically lacks the 18S and 28S ribosomal RNA peaks compared to the total RNA of its donor cell. Comparison of the RNA profiles has demonstrated that exosome-derived RNA contains mRNAs, lnRNAs, and circRNAs [50–52]. Thus, detecting the specific components secreted by tumor-derived exosomes, such as non-coding RNA, may possibly lead to the discovery of new cancer biomarkers.

Increasing evidence has demonstrated that the long RNA species have extensive clinical applications (Table 1). Researchers have found an absence of human telomerase reverse transcriptase (hTERT) mRNA in serum-derived exosomes compared to normal controls, despite being detectable in patients of different cancers [53]. In addition, recent studies have shown that the number of three long-chain RNAs (two mRNAs, KRTAP5–4, MAGEA3 and one lncRNA, BCAR) in serum exosomes of patients with colorectal adenoma is significantly different from healthy subjects, with the area under the ROC curve (AUC) being 0.936. Similarly, the detection of plasma-derived exosomal lncRNA HOTTIP may be a potential biomarker for diagnosing gastric cancer [54]. Its expression levels were significantly correlated with invasion depth (*P* = 0.0298) and TNM stage (*P* < 0.001). The AUC for exosomal HOTTIP was 0.827, which demonstrated a higher diagnostic capability than current tumor biomarkers, such as CEA, CA 19–9 and CA72–4 (AUC = 0.653, 0.685, and 0.639, respectively) (P < 0.001). Furthermore, there have been reports on the presence of circRNAs in exosomes [8], as well as certain circRNAs with varying expression patterns in normal and cancerous tissues [55]. Therefore, the long RNA species show great potential as future biomarkers and therapeutic targets. Further research will not only trigger the discovery of novel circulating biomarkers, but will also help researchers identify more molecular signatures of exosomes with functional applications.

**Table 1:** Exosomal long RNA species in cancer biology

Long RNA Species		Cancer Type	Biological Function	Mechanism	Refs
mRNA	ZEB1 mRNA	Non-small cell lung cancer	Transfer mesenchymal and chemoresistant phenotypes	Transfer of ZEB1 mRNA	[62]
	EGFRvIII mRNA	Glioblastoma	Diagnosis biomarkers	Unknown	[6]
	hTERT mRNA	Prostate cancer	Diagnosis biomarkers	Unknown	[53]
	KRTAP5–4 and MAGEA3	Colorectal adenoma	Diagnosis biomarkers	Unknown	[11]
	Annexin 2, Smad2, P27, MTAP, CIP4, and PEDF	Osteosarcoma	Biomarkers to classify osteosarcoma with different chemosensitivities	Unknown	[63]
lncRNA	Lnc-H19	Liver cancer	Promote angiogenesis	Increase the expression of VEGF, VEGFA, and ICAM	[43]
	lnc-CCAT2	Glioma	Promote angiogenesis and tumor progression	Activate VEGFA/ TGFβ and decrease apoptosis	[74]
	Linc-POU3F3	Glioma	Promote angiogenesis	Increase the expression of bFGF, bFGFR, VEGFA, and Angio	[75]
	Lnc-HULC	Pancreatic cancer	Promote invasion and metastasis	Inhibit E-cadherin mRNA expression and promote Snail and vimentin expression	[76]
	lncRNA-zfas1	Gastric cancer	Diagnosis biomarkers	Unknown	[77]
	LINC-ROR	HCC	Enhance drug resistance	Elevate TGF-β level	[80]
	Lnc-VLDLR	HCC	Enhance drug resistance	Strengthening ABCG2 expression to promote drug excretion from the cytoplasm	[81]
	lncARSR	Renal cancer	Enhance drug resistance	Act as a ceRNA for miR-34 and miR-449	[82]
	lncRNA-UCA1	Breast cancer	Enhance drug resistance	Decrease apoptosis	[83]
	lncRNA-p21	Prostate cancer	Suppress cancer initiation	Suppress genes regulated by p53	[88, 89]
	HOTAIR	Urothelial bladder cancer	Facilitate tumor initiation and progression	Regulate EMT associated genes	[90]
	MALAT1	Cervical cancer	Facilitate tumor progression	Modulate EMT	[91]
	MEG3A	Cervical cancer	Inhibit cell proliferation	Activate cell cycle arrest and apoptosis	[91]
	LINC00152	Gastric cancer	Diagnosis biomarkers	Unknown	[92]
	lncRNA-CRNDE-h	Colon cancer	Diagnosis biomarkers	Unknown	[93]
circRNA	Circ-CDYL	Liver cancer	Promote angiogenesis	Unknown	[8]
	Circ-KLDHC10	Colon cancer	Diagnosis biomarkers	Unknown	[8]
	CircFAT1	Colon cancer	Diagnosis biomarkers	Asssociated with the mutant KRAS	[115]
	CircRTN4	Colon cancer	Diagnosis biomarkers	Asssociated with the mutant KRAS	[115]

## Messenger RNA

Messenger RNAs (mRNAs) have been identified as important exosomal cargos and functional regulators of cell behavior in cancer-derived exosomes. Intriguingly, the state in which exosomes are released has different effects on the recipient cells. Using chip technology analysis, Eldh et al. found that exosomes secreted by cells under oxidative stress can result in recipient cells producing anti-oxidant activity as compared to those produced under normal conditions [56]. Exposing exosomes to UV light furthered weakened the protective effect of exosomes in shielding recipient cells from oxidative stress, resulting in cell death. This indicates that exosomes have the ability to communicate a protective signal to its recipient host cell and is a critical component of cell communication.

Seminal research by Valadi et al. determined the presence of exosomal mRNA and miRNA in mouse and human mast cells (MC/9 and HMC-1, respectively) by microarray analysis [50]. Assessments revealed that the distribution of mRNA in exosomes did not match with those found in the cytoplasm of donor cells, suggesting that mRNA and miRNA molecules are selectively loaded into exosomes. The distribution of mRNA in exosomes is significantly different in distinct cell types and species [51, 57]. Researchers found that specific mRNAs involved in cell migration, angiogenesis, and proliferation were present in glioblastoma-derived exosomes, whereas mRNAs associated with protein synthesis and cell development were detected in mouse-specific mast cells (MC/9). In addition, after translocation of murine mRNA into human mast cells, new murine proteins were found in the recipient cells. This implies that the transferred exosomal mRNA is functional after relocation and can be translated into proteins. Many researchers refer to this type of RNA as “exosomal shuttle RNA” (esRNA).

### Exosome-derived mRNAs in the cancer biology

Through network analyses, Hong et al. found that colorectal cancer (CRC) cell-derived exosomes are rich in cell cycle-related mRNAs that promote endothelial cell proliferation. Most of the EVs enriched with mRNAs were associated with M-phase activities, and were differentially regulated in CRC patients—suggesting that exosomes derived from cancerous cells may be involved in tumor growth and metastasis [46]. Additional studies have also identified the mRNA transport pathways of tumor-derived exosomes between different tumor cells [58, 59]. Tumor cell-derived exosomes were shown to be able to transfer mRNA to other types of tumor cells via clathrin-mediated endocytosis. This suggests that exosome delivery of mRNA may have therapeutic utility in diagnosing multiple organ tumorigenesis.

Exosome-derived mRNA can be additionally translated or transcribed into cDNA in recipient cells [60]. These features demonstrate an exosomal capability of affecting the expression of the target cell. Fluorescently labeled glioblastoma EVs were demonstrated to transfer Gaussia luciferase (Gluc) mRNA to human brain microvascular endothelial cells (HBMVECs), leading to the translation of proteins [6]. Tannous et al. used luciferase-encoding lentiviral vectors encoding Gluc to study the transport of RNA in exosomes [61]. Purified microbubbles containing Gluc mRNA were added to HBMVECs, and the activity of Gluc released into the medium was monitored over time. Gluc activity produced by the recipient cells continued to increase within 24 h, indicating that Gluc mRNA was undergoing translation. This shows that mRNAs incorporated into tumor EVs can produce functional proteins after being transported to recipient cells, facilitating a horizontal transfer of genetic information.

Lobb et al. demonstrated that exosomes derived from mesenchymal non-small cell lung cancer (NSCLC) cells have important functions in primary tumors by altering the phenotype of recipient cells [62]. The authors used a model of human bronchial epithelial cells (HBECs) in which donor cells were converted from the epithelium to the mesenchymal phenotype by introducing oncogenic changes commonly found in NSCLC. An increase of EMT-associated transcription factor ZEB1 mRNA was detected in the recipient cells, in addition to observing a transfer of chemoresistant phenotypes to donor epithelial HBECs through NSCLC exosomes. This work demonstrates for the first time that exosomes derived from oncogene-converted, mesenchymal lung cells may transfer mesenchymal and chemoresistant phenotypes into recipient cells through the transfer of ZEB1 mRNA.

### Outlook of mRNA biomarkers

Skog et al. showed that the mRNA found in serum-derived exosomes of glioblastoma patients could be translated into the mutein EGFRvIII [6]. In glioma patients, about 50% of the tumor mRNA mutations occur in patients with plasma exosomal mRNA mutations. In fact, no exosome-derived mutant mRNA was detected in the serum of patients who underwent surgical tumor resection, implying that exosomal mutant mRNA is derived from tumor cells. March-Villalba et al. additionally found that plasma telomerase reverse transcriptase (hTERT) mRNA was upregulated in the peripheral blood and tumor tissues of prostate cancer patients [53]. The size of the tumors was associated with the degree of malignancy—suggesting its potential as a non-invasive tumor marker in the diagnosis of prostate cancer.

Recent studies have shown that the number of long-chain RNAs, mRNAs KRTAP5–4 and MAGEA3 in serum-derived exosomes of colorectal adenoma patients is significantly different from healthy controls (AUC = 0.936) [11]. This further suggests that mRNAs may be used for the early detection of cancer. Researchers found that certain mRNAs such as Annexin 2, Smad2, P27, MTAP, CIP4, and PEDF, are differentially expressed in the exosomes of osteosarcoma patients under different chemotherapeutic responses [63]. These data indicate that exosomal mRNAs are reliable biomarkers for the classification of osteosarcoma with different chemosensitivities.

Nabet et al. showed that breast cancer cells trigger the NOTCH-MYC signaling pathway in tumor fibroblasts to promote the exosomal release of unshielded RN7SL1 RNA [64]. After the transfer of exosomes to immune cells, breast cancer cells, bone marrow, and dendritic cells, unshielded RN7SL1 triggered inflammatory responses, enhanced tumor growth, metastasis, and therapeutic resistance, in addition to inducing expression of CD40, CD86, PDL1, and MHCII. Evidence from tumor and blood samples confirmed that the activation of stromal cells results in the coupling of RNA damage-associated molecular patterns (DAMPs) and unshielded RNA to promote the aggressive features of cancer. Assessments of the blood samples of breast cancer patients revealed the presence of unshielded RN7SL1 in exosomes. Thus, exosomal content can be utilized to identify patients with more aggressive cancers by detecting exosome-binding RN7SL1.

## Long non-coding RNA

Long non-coding RNAs (lncRNAs) are RNA transcripts longer than 200 nucleotides that have limited or no protein-coding capacity. LncRNAs are involved in many important biological processes such as chromatin modification, gene expression, and nuclear transport, and are regulators of apoptosis, tumor migration, and drug resistance. Although lncRNA was once thought to be transcriptional noise, further investigation has demonstrated that lncRNAs play a functional role in carcinogenesis, tumor regulation, and gene expression [65, 66]. Its functional relevance in cancer also hints at the possibility of using lncRNAs as future diagnostic markers, drug delivery mediums, or targets for cancer treatments.

Exosome-derived lncRNAs have been detected in a wide range of bodily fluids due to active cellular secretion. Although ribonuclease is present in the blood, lncRNA nevertheless exists stably due to the protection of exosomes and microvesicles. Observation of the RNA content of exosomes, apoptotic bodies, and microvesicles in the blood showed that lncRNAs are mainly distributed in exosomes [11, 51]. This strongly implies that lncRNAs may be secreted into the bloodstream through this type of extracellular vesicle. Hewson et al. showed that certain exosome-related lncRNAs are involved in cellular metabolism, nucleosome structure, and cell signaling through interaction with L-lactate dehydrogenase B (LDHB), high-mobility group protein 17 (HMG-17), and CSF2RB [67]. These lncRNAs can bind to specific proteins, interact with candidate proteins, or be packaged into exosomes after being introduced into cells. Knowledge of the interactions of cellular pathways and exosome-associated non-coding transcripts can not only better clarify cell-to-cell interactions, but also provide a better understanding of the future of exosomal cell-targeted therapies.

### Exosome-derived lncRNAs in cancer biology

Exosomes are considered important mechanisms for the crosstalk between various cells. They have been shown to function as transport vesicles for functional lncRNA, which may affect the phenotype of the recipient cells [68–70]. In the tumor microenvironment, tumor cells and tumor-associated macrophages (TAMs) are reported as the most significant sources of exosomes. Interaction of ovarian cancer cells with TAMs can enhance endothelial cells’ ability to promote the development of ovarian cancer. However, how epithelial ovarian cancer (EOC) cells regulate the interactions between TAM and endothelial cells is still not clear. Wu et al. showed that TAM-derived exosomes could inhibit the migration of endothelial cells by targeting the miR-146b-5p/TRAF6/NF-kB/MMP2 pathway [71]. Yet, the addition of EOC-derived exosomes into the co-culture system restored the migration of endothelial cells by transferring lncRNAs to remotely reverse this effect. This suggests that lncRNAs have a powerful role in the regulation of the tumor microenvironment.

Previous studies have demonstrated that exosome-derived lncRNA can affect tumor growth, metastasis, invasion, and prognosis by regulating the tumor microenvironment [72, 73]. By traveling to cells through exosomes, lncRNAs can create a microenvironment suitable for the metastasis of tumor cells. Conigliaro et al. showed that the exosomes secreted by CD90 and tumor stem cells can induce an angiogenic effect in human umbilical vein endothelial cells (HUVECs) [43]. Through its adhesion to CD90 cells and HUVECs, such exosomes can invade endothelial cells, deliver lncRNA H19 to its corresponding target cells, and stimulate angiogenesis by synthesizing and releasing vascular endothelial growth factors (VEGF). Moreover, Lang et al. found that U87MG cells can also promote angiogenesis by transporting lnc-CCAT into endothelial cells through exosomes [74]. Secretion of exosomes rich in linc-POU3F3 induced angiogenesis in glioma cells by increasing the expression of basic fibroblast growth factor (bFGF), basic fibroblast growth factor receptor (bFGFR), VEGFA, and Angio [75]. Further investigations have also confirmed that the overexpression of lnc-CCAT2 upregulates the expression of VEGFA and TGFβ in HUVECs—reducing apoptosis by promoting angiogenesis and Bcl-2 expression and inhibiting Bax and caspase-3 expression.

In a study by Takahashi and colleagues, TGF-β induced interstitial epithelial cells and promoted the invasion and metastasis of cancer cells [76]. TGF-β induced the upregulation of several lncRNAs in pancreatic cells, of which lncRNA-HULC (lnc-HULC) demonstrated the most prominent changes. After lnc-HULC knockdown, the survival, invasion, and migration abilities of the cells decreased, but the level of lnc-HULC in exosomes derived from TGF-β-induced pancreatic cells increased. LncRNA zfas1 was additionally found to be related to lymphatic metastasis in gastric cancer patients [77]. Its transmission through exosomes enhanced cell proliferation and migration of cancerous cells—strongly indicating that tumors can enhance cellular migration and invasion abilities through exosome-derived lncRNAs.

### Exosome-derived lncRNA and chemoresistance

Drug resistance is often the main reason for the failure of clinical chemotherapy. Exosome-mediated translocation of non-coding RNAs have been shown to be an important mechanism of acquired drug resistance in some cancer cells [78, 79]. Long intergenic non-coding RNA ROR (LINC-ROR) was found to be an effector molecule of chemotherapeutic drug resistance in hepatocellular cancer cells (HCC) [80]. High expression of LINC-ROR was detected in HCC cells, but more specifically, they were enriched in the extracellular vesicles derived from tumor cells. An increased level of chemoresistance was observed in HCC cells treated with exosomes containing high levels of LINC-ROR. Similarly, the cells’ sensitivity to chemotherapy increased after LINC-ROR knockdown. As hepatocellular cancers are known to be highly resistant to chemotherapy, this suggests that cancerous cells may be utilizing exosome-derived lncRNAs to improve chemoresistance.

Another study found that HCC cells exposed to different chemotherapy drugs, such as sorafenib, camptothecin, and doxorubicin, increased the expression of lnc-VLDLR in cells and the exosomes secreted from these cells [81]. Expression profiling identified that lnc-VLDLR was significantly upregulated in cancerous cells. Recipient cells co-cultured with these exosomes reduced chemotherapy-induced cell death and increased lnc-VLDLR expression in the recipient cells. Takahashi et al. concluded that lnc-VLDLR was an lncRNA-rich extracellular vesicle that contributed to cellular stress response.

Recently, lncRNA activated in RCC with sunitinib resistance (lncARSR) was found to be an endogenous competing RNA that promotes sunitinib resistance in resistant renal tumor exosomes. In sunitinib-resistant renal tumors, overexpressed lncARSR competes with miR-34 and miR-449, promotes AXL and c-MET expression, reactivates RTKs, and mediates drug resistance [82]. Exosomes of tamoxifen-resistant LCC2 cell lines were additionally found to contain high levels of lncRNA-UCA1 [83]. Exosome-mediated transport of UCA1 significantly increased tamoxifen resistance in ER-positive MCF-7 cells. MCF-7 cells pretreated with ExoS/Lcc2 significantly increased cell viability, decreased the expression of cleaved caspase-3, and reduced apoptosis rates after treatment with tamoxifen. These findings provide new insight into the role of exosomal lncRNA and demonstrate the mediating ability of lncRNA in chemoresistance.

### Exosome-derived lncRNAs as biomarkers

Exosomal lncRNAs have strong clinical application prospects as it can be used in many common experimental techniques such as real-time fluorescent quantitative polymerase chain reaction (RTFQ-PCR), gene chip analysis, and other sequencing tests [84, 85]. In addition, they can be conveniently drawn and are stable in nature [86, 87]. Although exosome-derived lncRNAs are pathophysiologically significant, the exact function of these lncRNAs remains unknown. However, these flaws in the understanding of its function or pathophysiological effect do not limit its possibility as a diagnostic biomarker.

With the increasing amount of studies on exosome-derived lncRNA, its application in other bodily fluids as a diagnostic clinical marker is of great interest. Isin et al. found that lncRNA-p21 expression is upregulated in benign prostate cancer compared to that of prostatic hyperplasia in exosomes extracted from the urine, suggesting that it can be used as a molecular marker for the differential diagnosis of prostate cancer [88, 89]. Similarly, researchers found that lncRNA HOX transcript antisense RNA (HOTAIR) was increased in urine-derived exosomes of urothelial bladder cancer (UBC) patients [90]. Specific knockdown of HOTAIR in vitro inhibited the migration and invasion abilities in UBC cell lines and altered the expression of genes involved in epithelial-mesenchymal transition (EMT), such as SNAI1, TWIST1, ZEB1, ZO1, MMP1, LAMB3, and LAMC2. These data indicate that urine-derived lncRNAs demonstrate a possibility as future therapeutic targets due to its extensive effect on the tumor microenvironment.

Furthermore, studies on exosome-derived lncRNAs in vaginal lavage specimens revealed that the expression of HOTAIR, metastasis associated lung adenocarcinoma transcript 1 (MALAT1), and maternally expressed 3 A (MEG3A) in cervical cancer patients and healthy samples were significantly different [91]. Similarly, LINC00152 was found to be highly expressed in the plasma-derived exosomes in gastric cancer patients. The expression level of LINC00152 in the plasma was not significantly different from that of plasma-derived exosomes—suggesting that LINC00152 is stably protected by exosomes. The diagnostic sensitivity of exosome-derived LINC00512 was 48.1%, specificity was 85.2%, and the AUC was 0.66—demonstrating a good diagnostic advantage [92]. In addition, the increase of lncRNA-CRNDE-h in the serum can be used to identify patients with colorectal cancer, benign colorectal disease, or healthy controls. The diagnostic sensitivity and specificity were 70.3% and 94.4%, respectively, and the AUC was 0.89 [93]. The applicability of lncRNAs in a wide range of cancers demonstrates its strong potential as convenient, noninvasive biomarkers for future cancer therapies.

### Prospects for exosome-derived lncRNAs

Due to its length, lncRNA can bind to both mRNA and miRNA at the same time, enabling it to play a silencing role in gene expression. Current research shows that its biological mechanism is extremely complex and may include the encoding of proteins [94, 95]. As such, lncRNAs may be the bridge between other non-coding RNA interactions. In 2016, Ahadi et al. found that four types of exosomes derived from prostate cancer cells contained certain lncRNAs [96]. These lncRNAs by nature are enriched with miRNA seed sequences, including let-7 family members as well as miR-17, miR-18a, Mir-20a, miR-93, and miR-106b. These exosomal lncRNAs also contained binding sequences for RNA binding proteins (RBP)—the two most common motifs being ELAVL1 and RBMX. Given that exosomal lncRNAs are rich in miRNA seed sequences and RBP sites, their interaction may play an important role in the carcinogenesis of cancer.

Despite its large potential, the study of lncRNAs in exosomes is still in its infancy. Yet, lncRNAs are ideal biomarkers because of their: 1) Specificity: Exosomes have specific markers of the tissue or cell of origin [97, 98], and the contents of exosomes released vary under different physiological or pathological conditions. Research has shown that despite a low cellular expression of lncRNA, they are highly expressed in exosomes—suggesting a selective loading mechanism for lncRNAs [99]. 2) Stability: Due to protection from the lipid bilayer, enzymes cannot easily digest the contents of exosomes. RNA levels did not change significantly after exposure to a variety of cellular stress conditions—indicating that external conditions have little effect on the stability of lncRNAs [100, 101]. 3) Accessibility: Researchers have been able to ensure rapid and accurate isolation of exosomes widely distributed in various types of body fluids through techniques such as flow cytometry, Western Blotting, and real-time PCR. Finally, due to the relatively stable contents of exosomes, studies have been applied to gene therapy. Shtam et al. transfected siRNA into exosomes, and successfully achieved the silencing of RAD51 using exosomes as vectors [102]. Thus, the emerging field of exosomal lncRNAs is worth our time and effort.

## Circular RNA

Circular RNAs (circRNAs) are a class of non-coding RNAs that have been recently found to be biologically functional in mammals [103–106]. Formed by backsplicing events where a downstream 5′ site binds to an upstream 3′ site, circRNAs lack 5′ and 3′ terminal ends [107]. As such, circRNAs are inherently resistant to the major enzymes of degradation, which work by targeting the 5′ and 3′ termini. This results in the high cellular stability and extended half-life often seen in circRNAs. CircRNAs were first reported in viruses as early as the 1970s, but have long been assumed to be RNA splicing errors [108, 109]. However, with the recent development of high-throughput sequencing, circRNAs have been shown to exist in large numbers of eukaryotic transcriptomes [105, 106, 110, 111]. Researchers detected more than 400 circRNAs in human saliva supernatants and found that the parental genes of these circRNAs are associated with inflammatory responses, cytoskeletal formation, cell motility, T cell polarity formation, and integrin-mediated signaling [112]. Despite this, the understanding of how cells regulate circRNAs is still limited. CircRNAs are largely reported as miRNA sponges that affect downstream target genes of miRNAs, regulate alternative splicing, and influence host gene transcription [105, 113, 114]. Further investigation is still needed to elucidate the precise function of circRNAs.

### Exosome-derived circRNAs in cancer biology

Li et al. demonstrated that circRNAs exist in exosomes through RNA sequencing analyses of hepatic MHCC-LM3 cancer cells and cell-derived exosomes [8]. More than 90% percent of the analyzed circRNAs were composed of exons, demonstrated high stability, and were not susceptible to exonuclease cleavage—signs of a possible tumor diagnostic marker. In a comparison of healthy donors and CRC patients, 67 circRNAs were absent and 257 new circRNAs were detected in cancer patients. In addition, 1215 exosomal circRNAs were derived from the human serum, with some circRNAs demonstrating significant differences in exosome content between CRC patients and normal controls. Further overexpression of miR-7 in HEK293T cells and MCF-7 cells showed that circRNA CDR1as was significantly downregulated in exosomes, suggesting that the process of circRNAs entering exosomes may be regulated by intracellular miRNAs.

Dou et al. found that the circRNA of the KRAS mutant (DKO-1) and the combined mutant/wild type (DLD-1) expressed lower abundance levels compared to that of the wild type (DKs-8) [115]. This suggests that the oncogene mutation reduces the expression of circRNA in cells. Exosomes in seven of the most abundant circRNAs of the wild type (DKs-8) were also highly expressed, indicating that circRNAs can be transferred from cells to exosomes. Similarly, Beckler et al. also found that the composition of the exosome proteome was significantly affected by mutated KRAS [116]. A comprehensive proteomic analysis of exosomal content showed that exosomes from mutated KRAS cells contain many tumor-promoting proteins that can be transferred to recipient cells.

Currently there are two hypotheses about the function of circRNA in exosomes: cell-to-cell communication and circRNA clearance. As exosomes are widely accessible in the bodily fluids, exosomes containing circRNAs have been shown to transfer biological activity to recipient cells. Li et al. found that circRNAs retained biological activity after being translocated into recipient cells as exosomes containing CDR1as inhibited miR-7 induced growth suppression [8]. Yet, Lasda et al. proposed that the presence of circRNAs in exosomes and extracellular vesicles is the mechanism by which cells clear endogenous circRNA [117]. The authors analyzed the relative amounts of circRNA and linear RNA in secreted extracellular vesicles in Hela, 293 T, and U2OS cells and found that the ratio of circRNA and linear RNA significantly increased. The authors proposed that secretory vesicles, including exosomes, are the mechanism by which cells selectively release endogenous circRNA. Since other cells, such as macrophages, absorb secreted vesicles, these vesicles may also act as a means of cell-to-cell communication. Is it possible that the presence of exosomal exonucleases lead to a decreased amount of linear RNA and an increased amount of circRNA? According to Alhasan et al., the phenomenon of platelet-rich circRNA is the result of exonucleases [118]; therefore we cannot completely rule out this possibility with the data presented in this article. Nonetheless, both hypotheses are relatively new and are worthy of further verification.

### Potential of circRNA in future treatments

Researchers report that circRNAs are not only located in cell-derived exosomes; in fact, a large number of complete and stable circRNAs are contained in tumor-derived exosomes—indicating that exosomal circRNA is highly anticipated as a future biomarker for tumor detection. A recent study showed that ciRS-7 expression has the capability to predict microvascular invasion (MVI) in HCC [119]. Xu et al. found that the expression levels of ciRS-7 are significantly correlated with the three clinicopatholgical parameters of HCC associated with the deterioration of the disease: age, serum levels, and hepatic MVI. This suggests that ciRS-7 may be a promising biomarker of hepatic MVI and a novel therapeutic target for restraining MVI in HCC.

CircRNAs have several advantages as potential therapeutic targets for future clinical applications. Tay et al. investigated the design and effects of different mRNA sponge expression vectors in malignant melanoma cell lines, and found that circular carriers can make mRNA sponges more durable and stable than linear ones [120]. Since circRNAs are not sensitive to nucleases, this may explain why circRNAs are more stable than linear RNAs. Because of its special circular structure, circular RNA sponges can add more specific miRNA binding sites to circRNAs, thus indefinitely enhancing their anti-miRNA ability [121]. Compared to linear RNA sponges, circRNA sponges may have a more potent inhibitory effect on the oncogenic activity of miRNAs because distinct circRNAs contain many specific miRNA-binding sites. In addition, they can indirectly regulate the expression of genes by affecting effector molecules in the miRNA pathway, thus acting as a miRNA sponge in different species [122, 123]. In the near future, it is believed that miRNA circular sponges are expected to become a new strategy for future RNA gene therapies targeted against cancer. As a stable and efficient miRNA inhibitor, artificial miRNA “sponge” technology may be a new alternative to gene knockdown. It can simultaneously inhibit the expression of other paralogous miRNAs, in addition to producing a more long-lasting inhibiting effect.

Currently, the understanding of the role of exosomal circRNA in malignancies, molecular mechanisms, and possible clinical applications are still limited and need further study. Databases for circRNAs have not yet been fully established, and the lack of an internationally accepted nomenclature for classification remains a problem to be solved. However, the establishment and development of exosomal databases, such as ExoCarta, ExAtlas, and exoRBase, will further facilitate these studies on the long RNA species.

## Available databases for exosomal RNA

With the recent exponential increase of exosomal research in the past decade, many exosome databases have been established (Table 2). Vesiclepedia is one of these databases, where it features active involvement of EV researchers in creating a continuous and updated database. It not only includes molecular information on exosomes, but also provides information on other classes of EVs, such as microparticles, microvesicles, and apoptotic blebs. Vesiclepedia currently consists of a total of 35,264 protein, 18,718 mRNA, 1772 miRNA, and 342 lipid entries from 341 independently published studies [124, 125]. Researchers are able to browse and retrieve information based on organism, vesicle type, content type, and sample material to search for related datasets, genes, or published studies. EVpedia is another comprehensive database that integrates high-throughput datasets of vesicular components (proteins, mRNAs, miRNAs, and lipids) from both prokaryotic and eukaryotic extracellular vesicles. At present, it includes 443 high-throughput studies, 957 high-throughput datasets, and 592,870 molecules [126–128]. It focuses on providing analytical tools for comparative analyses, such as ortholog identification, Gene Ontology enrichment analyses, and network analyses.

**Table 2:** Available exosomal RNA databases

Name	Content	Features	Website
Vesiclepedia	Extracellular vesicles (protein, mRNA, miRNA, and lipids)	Different Classes of EVs	http://microvesicles.org/
EVpedia	Prokaryotes & eukaryotic extracellular vesicle components (proteins, mRNAs, miRNAs, and lipids)	EV Markers	http://evpedia.info
ExoCarta	Exosomal proteins,mRNA, miRNAs, and lipids	Exosome Markers	http://www.exocarta.org/
exRNA Atlas	exRNA	RNA Profiles	http://exrna-atlas.org/
miRandola	Non-coding RNA	miRNA Converter	http://mirandola.iit.cnr.it/
ExoRBase	Exosomal long RNA species (circRNAs, lncRNAs, and mRNAs)	Focuses on human blood exosomes	http://www.exorbase.org

Several databases have been developed solely for exosomes and extracellular biomarkers. For example, ExoCarta contains molecular information only on exosomes. The ExoCarta database is an open platform that contains more than 286 studies on proteins, mRNAs, miRNAs, and lipids in exosomes derived from different tissues and organs [129–131]. It features dynamic protein-protein interaction networks and biological pathways of exosomal proteins, which can be imported into FunRich, a tool for additional enrichment and analysis. ExRNA Atlas is another database that extracts exRNA profiles from human and mouse biofluids generated by the Extracellular RNA Communication Consortium (ERCC) [132–134]. It is an integrative software that analyzes and visualizes datasets in the context of specific biological pathways and networks, in addition to providing standardized exRNA protocols. Other more specialized databases, such as miRandola, can be used to study the biological functions of predicted extracellular biomarkers in its circulating non-coding RNA database [135, 136].

The establishment of exoRBase has recently drawn the attention of many researchers, as it focuses on the long RNA species specifically derived from human blood exosomes. It currently contains 18,333 mRNAs, 15,501 lncRNAs, and 58,330 circRNAs, in addition to providing its expression level, annotation, and possible tissues of origin [10]. It integrates and visualizes RNA expression profiles based on normalized RNA-sequence samples of healthy controls and patients of different diseases. With the development of these exosomal databases, it is certain that the functional implications and mechanisms of exosomes will be gradually elucidated—advancing the discovery of exosomal biomarkers and therapeutic targets.

## Conclusions

Despite exosomes being regarded as waste cargo when initially discovered [21, 22], this perception changed when RNA content was detected in exosomes in 2007 [50]. From the discovery of RNA content in exosomes to the first studies published on exosomal RNA as biomarkers [6, 8, 9, 70], and finally to the creation of exosomal databases [10, 131], exosome-derived RNA have been the spotlight of exosomal research. A timeline of the significant events of exosomal RNA research is presented in Additional file 2. As we have witnessed in this past decade, exosomes are emerging as promising tools for the treatment and diagnosis of cancer. Their applicability, accessibility, and specificity are factors that make them attractive targets for researchers. However, despite the advances in exosome detection, there are some limitations in the study of exosomes. For instance, a standardized method for collecting, handling, and isolating exosome samples has yet to be established [137, 138]. The present methods of extracting exosomes are extremely time-consuming and not practical for routine diagnostics. The purity of tumor-derived exosome samples is another issue, as samples collected from the serum and plasma may include EVs released by cells other than tumor cells [139]. Improvements to the current strategies are needed to advance research on exosomes.

Successful treatment of cancer depends on the understanding of the complex mechanisms in the tumor microenvironment. Exosomes, as critical components of cellular communication, function as key facilitators in the exchange of information between cells. The accessibility of exosomes in nearly all biofluids indicates its unprecedented possibility in a wide range of cancers, as demonstrated by its extensive impact on the tumor microenvironment. In addition, their ability to mediate cell communication makes them strong targets for future therapeutic treatments as well as potential vectors for drug delivery. Exosomal contents, namely mRNAs, lncRNAs, and circRNAs, can reflect the progression of disease—highlighting their importance as promising, non-invasive biomarkers for diagnostic and prognostic purposes. Further studies on exosomes will not only have profound impacts on the treatment of cancer, but will also lay the foundation for novel treatment methods employing exosomes as biomarkers and therapeutic targets.

## Additional files

Additional file 1: Exosomal RNA articles published per year. Graph showing the number of articles published relating to exosome-derived RNA per year since 2007. (DOCX 58 kb)

Additional file 2: Timeline. A timeline of the important discoveries in exosome research, focusing on the breakthroughs in exosomal RNA research. (PPTX 90 kb)

## Abbreviations

AUC: Area under the ROC curve
bFGF: Basic fibroblast growth factor
bFGFR: Basic fibroblast growth factor receptor
circRNA: Circular RNA
CRC: Colorectal cancer
DAMPs: Damage-associated molecular patterns
EMT: Epithelial-mesenchymal transition
EOC: Epithelial ovarian cancer
ERCC: Extracellular RNA Communication Consortium
esRNA: Exosomal shuttle RNA
EV: Extraceullular vesicle
Gluc: Gaussia luciferase mRNA
HBEC: Human bronchial epithelial cell
HBMVEC: Human brain microvascular endothelial cells
HCC: Hepatocellular carcinoma
HMG-17: High-mobility group protein 17
HOTAIR: lncRNA HOX transcript antisense RNA
hTERT: Human telomerase reverse transcriptase
HUVECs: Human umbilical vein endothelial cells
LDHB: L-lactate dehydrogenase B
LINC-ROR: Long intergenic non-coding RNA ROR
lncARSR: lncRNA activated in RCC with sunitinib resistance
lnc-HULC: lncRNA-HULC
lncRNA: Long non-coding RNA
MALAT1: Metastasis associated lung adenocarcinoma transcript 1
MEG3A: Maternally expressed 3 A
miRNA: MicroRNA
mRNA: Messenger RNA
MVB: Multi-vesicular body
MVI: Microvascular invasion
NK: Natural killer cells
NSCLC: Non-small cell lung cancer
RBP: RNA binding proteins
RTFQ-PCR: Real-time fluorescent quantitative polymerase chain reaction
TAMs: Tumor-associated macrophages
UBC: Urothelial bladder cancer
VEGF: Vascular endothelial growth factors
