# Supplementary material for: The decade of exosomal long RNA species: an emerging cancer antagonist
Source: Mol Cancer. 2018 Mar 20;17:75. doi: 10.1186/s12943-018-0823-z (PMC5861621; doi:10.1186/s12943-018-0823-z)
Supplement: Supplementary file 1 — Exosomal RNA articles published per year. Graph showing the number of articles published relating to exosome-derived RNA per year since 2007. (DOCX 58 kb) [file 12943_2018_823_MOESM1_ESM.docx]

**Additional file 1.** Exosome articles published per year. Graph showing the number of articles relating to exosome-derived RNA per year since 2007. “Exosome RNA” was entered in the search box of PubMed at the NCBI webpage. Limits were set by searching one year at a time. Articles published ahead of print for 2018 were not included.
