# Supplementary material for: The decade of exosomal long RNA species: an emerging cancer antagonist
Source: Mol Cancer. 2018 Mar 20;17:75. doi: 10.1186/s12943-018-0823-z (PMC5861621; doi:10.1186/s12943-018-0823-z)
Supplement: Supplementary file 2 — Timeline. A timeline of the important discoveries in exosome research, focusing on the breakthroughs in exosomal RNA research. (PPTX 90 kb) [file 12943_2018_823_MOESM2_ESM.pptx]

## Slide 1
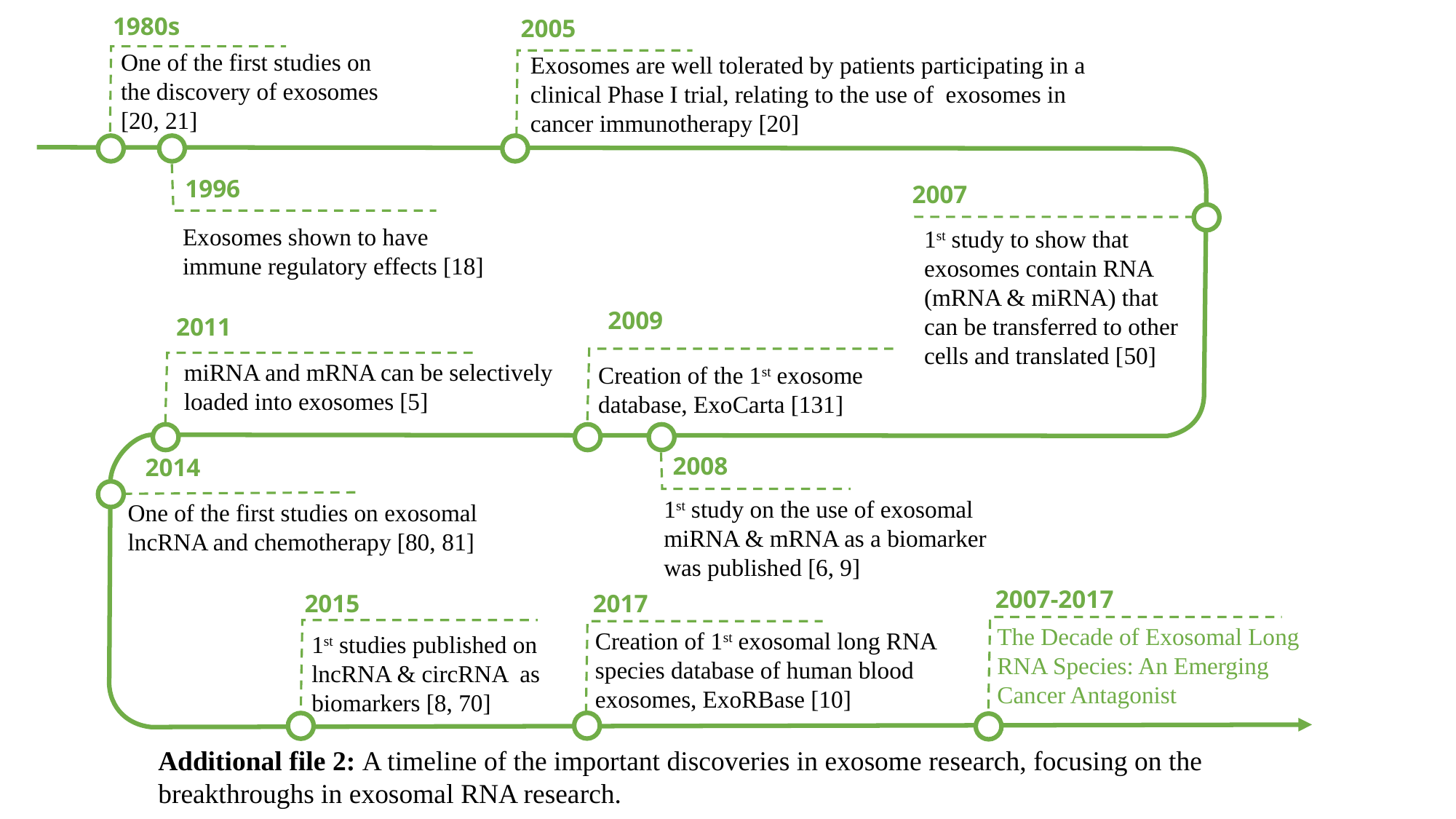

1980s
One of the first studies on the discovery of exosomes [20, 21]
2005
Exosomes are well tolerated by patients participating in a clinical Phase I trial, relating to the use of exosomes in cancer immunotherapy [20]
1996
Exosomes shown to have immune regulatory effects [18]
2007
1st study to show that exosomes contain RNA (mRNA & miRNA) that can be transferred to other cells and translated [50]
2009
Creation of the 1st exosome database, ExoCarta [131]
2011
miRNA and mRNA can be selectively loaded into exosomes [5]
2008
1st study on the use of exosomal miRNA & mRNA as a biomarker was published [6, 9]
2014
One of the first studies on exosomal lncRNA and chemotherapy [80, 81]
2015
1st studies published on lncRNA & circRNA as biomarkers [8, 70]
2017
Creation of 1st exosomal long RNA species database of human blood exosomes, ExoRBase [10]
The Decade of Exosomal Long RNA Species: An Emerging Cancer Antagonist
2007-2017
Additional file 2: A timeline of the important discoveries in exosome research, focusing on the breakthroughs in exosomal RNA research.
